# Very early vs delayed invasive strategy in high-risk NSTEMI patients without hemodynamic instability: Insight from the KAMIR-NIH

**DOI:** 10.1371/journal.pone.0304273

**Published:** 2024-06-06

**Authors:** Seung Do Lee, Rock Bum Kim, Chang-Ok Seo, Moojun Kim, Hyo Jin Lee, Hangyul Kim, Hye Ree Kim, Kyehwan Kim, Min Gyu Kang, Jeong Rang Park, Suk Jae Hwang, Jin Yong Hwang, Myung Ho Jeong, Seung-Ho Hur, Kwang Soo Cha, Jin-Sin Koh, on behalf of the KAMIR-NIH registry investigators

**Affiliations:** 1 Division of Cardiology, Department of Internal Medicine, Gyeongsang National University School of Medicine, Gyeongsang National University Hospital, Jinju, Korea; 2 Department of Preventive Medicine and Institute of Health Sciences, Gyeongsang National University College of Medicine, Jinju, Korea; 3 Chonnam National University Hospital, Gwangju, Republic of Korea; 4 Keimyung University Dongsan Medical Center, Cardiovascular Medicine, Deagu, Republic of Korea; 5 Pusan National University Hospital, Busan, South Korea; Saud Al-Babtain Cardiac Centre, SAUDI ARABIA

## Abstract

**Background:**

High-risk non-ST-elevation myocardial infarction (NSTEMI) patients’ optimal timing for percutaneous coronary intervention (PCI) is debated despite the recommendation for early invasive revascularization. This study aimed to compare outcomes of NSTEMI patients without hemodynamic instability undergoing very early invasive strategy (VEIS, ≤ 12 hours) versus delayed invasive strategy (DIS, >12 hours).

**Methods:**

Excluding urgent indications for PCI including initial systolic blood pressure under 90 mmHg, ventricular arrhythmia, or Killip class IV, 4,733 NSTEMI patients were recruited from the Korea Acute Myocardial Infarction Registry-National Institutes of Health (KAMIR-NIH). Patients were divided into low and high- global registry of acute coronary events risk score risk score (GRS) groups based on 140. Both groups were then categorized into VEIS and DIS. Clinical outcomes, including all-cause death (ACD), cardiac death (CD), recurrent MI, and cerebrovascular accident at 12 months, were evaluated.

**Results:**

Among 4,733 NSTEMI patients, 62% had low GRS, and 38% had high GRS. The proportions of VEIS and DIS were 43% vs. 57% in the low GRS group and 47% vs. 53% in the high GRS group. In the low GRS group, VEIS and DIS demonstrated similar outcomes; however, in the high GRS group, VEIS exhibited worse ACD outcomes compared to DIS (HR = 1.46, P = 0.003). The adverse effect of VEIS was consistent with propensity score matched analysis (HR = 1.34, P = 0.042).

**Conclusion:**

VEIS yielded worse outcomes than DIS in high-risk NSTEMI patients without hemodynamic instability in real-world practice.

## 1. Introduction

The TIMACS trial, a pivotal randomized controlled trial (RCT) designed to determine the optimal timing of percutaneous coronary intervention (PCI) in patients with non-ST-segment elevation myocardial infarction (NSTEMI), revealed that while adopting an early invasive strategy (EIS) with coronary angiography (CAG) within 24 hours did not show significant differences compared to a delayed invasive strategy (DIS) performed after 36 hours, EIS yielded more favorable outcomes, especially in patients at high-risk as identified by a global registry of acute coronary events risk score risk score (GRS) exceeding 140 (1). In addition to TIMACS, several other RCTs have consistently reported that EIS does not inherently result in overall improved clinical outcomes in patients with NSTEMI, except for a notable reduction in patients with high-risk profiles [1–7]. Based on these findings, the current guidelines recommend implementing EIS within 24 hours for NSTEMI patients with high-risk scores [8, 9].

However, despite these guidelines, the optimal timing of PCI in patients with NSTEMI based on individual risk factors remains controversial. The recent post hoc analysis of VERDICT trial presented contrasting results pertaining to the timing of PCI in patients with NSTEMI. The trial found that a very early invasive strategy (VEIS) within 12 h was associated with an increased risk of mortality in those with a low GRS (≤140), while indicating a reduced risk of mortality in those with a high GRS (>140) [10] These divergent outcomes from various trials highlight the need to carefully consider the ideal timing for PCI in patients with NSTEMI, excluding cases in which urgent PCI is required due to hemodynamic instability resulting from cardiac collapse. A recent real-world analysis of cohorts comprising of patients with NSTEMI demonstrated that VEIS within 12 hours was not linked to improved one-year cardiovascular outcomes, even in high-risk cases. This was compared to delayed angiography performed between 12 and 24 h after admission [11].

Therefore, we investigated the impact of a very early invasive strategy (VEIS) within 12 hours of admission and compared it with a delayed invasive strategy (DIS) after 12 hours of admission on clinical outcomes in real-world patients with NSTEMI using a nationwide, multicenter, large cohort with acute myocardial infarction (AMI) patients, considering varying risk levels (low and high GRS).

## 2. Materials and methods

### 2.1 Patient population

The KAMIR-NIH is a nationwide, multicenter prospective registry designed to assess clinical outcomes in patients experiencing AMI from 20 university hospitals in South Korea between November 2011 and December 2015 [12]. During this period, a total of 13,104 patients were enrolled. Patients aged > 18 years who were diagnosed with AMI based on symptoms, cardiac markers, and electrocardiographic changes in ST-segment elevation myocardial infarction (STEMI) or NSTEMI were included. Of these patients, 5,652 fulfilled the NSTEMI criteria, underwent PCI for revascularization and were included in this study. After excluding those with unavailable GRS measurements or those requiring urgent PCI due to severe hypotension (SBP <90 mmHg), ventricular arrhythmia, or Killip class IV, the final analysis included 4,733 patients with NSTEMI who underwent PCI. Patients were divided into two groups based on the GRS score: low GRS (≤ 140) and high GRS (> 140). In line with current international guidelines and standard clinical practice, an invasive strategy for NSTEMI was defined as referring for CAG, regardless of any subsequent coronary interventions. The timing of intervention was characterized as the duration between hospital arrival and coronary angiography. Each group was subsequently divided into VEIS and DIS, based on the 12-hour framework for PCI timing. Data for the research were gathered using an online case report form at each of the participating centers. The study adhered to the ethical principles outlined in the 2004 Declaration of Helsinki and received approval from the ethics committees at each participating center, as well as from the Chonnam National University Hospital Institutional Review Board (CNUH-2011-172). Prior to their participation, all patients in the study provided written informed consent. They were monitored through various means such as face-to-face interviews, phone calls, and chart reviews, during follow-up periods. An independent committee was responsible for assessing and evaluating all clinical events.

### 2.2 Study definitions and clinical outcomes

NSTEMI was defined as clinical symptoms and ECG findings consistent with acute myocardial ischemia, except ST elevation, presenting with an increase in cardiac troponin I or T. A successful PCI was defined as residual stenosis of < 30% and thrombolysis in myocardial infarction (TIMI) flow grade 3 in the infarct-related artery (IRA). The GRS was calculated based on eight independent risk factors: age, heart rate, systolic blood pressure, Killip class, cardiac arrest, ST segment deviation, serum creatinine level, and initial cardiac biomarker status [1]. CAG and PCI were performed following general PCI guidelines at the discretion of individual operators based on each patient’s specific condition. Prior to PCI, patients were prescribed conventional dual antiplatelet therapy (DAPT), including aspirin and P2Y12 inhibitors (clopidogrel, ticagrelor, or prasugrel), as a loading dose, and all patients were guided to take a maintenance dose after the procedure. Optimal medical treatment (OMT) is the use of a combination of DAPT, renin-angiotensin-aldosterone system inhibitors (RAASi), beta-blockers (BB), and statins. The extent of coronary artery disease (CAD) was classified as single-vessel disease (SVD), multi-vessel disease (MVD) containing more than two vessel diseases, and single-left main (LM) CAD. The primary clinical outcome was death due to any cause during the 12-month follow-up period. The secondary outcomes were in-hospital mortality, cardiac death (CD), recurrent myocardial infarction (MI), and cerebrovascular accident (CVA) at the 12-month follow-up.

### 2.3 Data collection

Data were collected by a trained study coordinator using a standardized case-report form and protocol. The patients were assessed at the outpatient clinic of the cardiology department at the end of the first month and every 6 months for 12 months. Clinical events, including cardiac or non-cardiac death, recurrent MI, and CVA, were recorded.

### 2.4 Statistics

For continuous variables, differences between groups were evaluated using the unpaired t-test or Mann-Whitney rank-sum test. For discrete variables, differences were expressed as counts and percentages, and they were analyzed with χ2 (or Fisher exact) test between groups as appropriate.

The cumulative incidence of clinical outcomes was determined using Kaplan-Meier analysis, and group differences were assessed using the log-rank test. Continuous variables, which were not normally distributed, were presented as median and IQR (25th-75th percentiles) values, with significance assessed using the Kruskal-Wallis test. The Hazard ratios (HR) and its 95% confidence intervals (CI) were computed using Cox proportional hazards regression analysis. Covariates deemed clinically relevant were incorporated to compute the HR and 95% CI after adjusting for multiple variables. These variables included dyslipidemia, diabetes, extent of CAD, serum hemoglobin level, optimal medical therapy, and revascularization status. To reduce errors resulting from variations in the size of the study population, we conducted propensity score-matching (PSM) analysis using a logistic regression model. We tested all accessible variables that may have potential significance, including baseline clinical characteristics and procedural factors (i.e., age, sex, systolic blood pressure [SBP], smoking, diabetes, dyslipidemia, serum Hb level, serum creatinine level, left ventricular ejection fraction (LVEF), revascularization status, OMT, and extent of CAD). Patients in the VEIS group were paired with individuals in the DIS group in a 1:1 ratio based on their propensity scores using the nearest available pair-matching technique. The matching process utilized a caliper width of 0.1, leading to 1,163 closely matched pairs for the low-GRS group and 747 closely matched pairs for the high-GRS group (S2 and S3 Tables). The C-statistics for PSM in this study were 0.822 and 0.744 for the low and high GRS groups, respectively. All analyses were 2-tailed, and clinical significance was defined as P < 0.05. Statistical analysis was performed using R software (version 4.2.1; R Foundation for Statistical Computing).

## 3. Results

### 3.1 Study population and baseline characteristics

**Fig 1:**
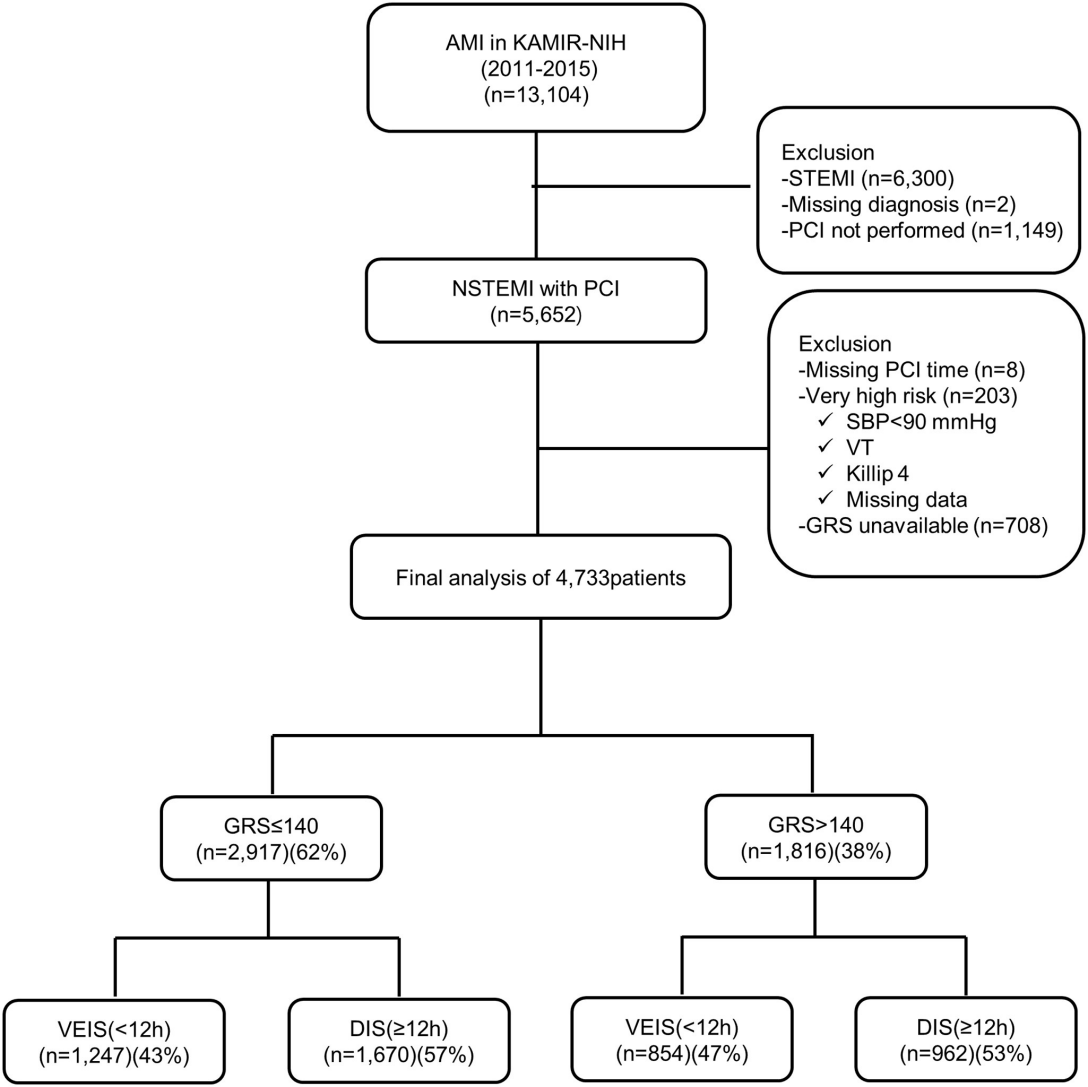
Flow chart of study design. AMI, acute myocardial infarction; KAMIR-NIH, Korea Acute Myocardial Infarction Registry-National Institute of Health; NSTEMI, non-ST elevation myocardial infarction; PCI, percutaneous coronary intervention; GRS, Global Registry of Acute Coronary Events risk score; VEIS, very early invasive strategy; DIS, delayed invasive strategy.

In a total of 4,733 patients with NSTEMI who underwent PCI, 62% (n = 2,917) had a low GRS (≤140), and 57% (n = 1,816) had a high GRS (>140) score. Among those with low GRS, VEIS was applied in 1,247 patients (43%), and DIS was applied in 1,670 patients (57%). However, in the high GRS group, a ratio of 47% versus 53% was applied, resulting in 854 patients for VEIS and 962 patients for DIS (Fig 1, Table 1). The median PCI time in the VEIS group was 3.8 hours, whereas it was 24.1 hours for the DIS group. Compared to the high GRS group, the low GRS group was younger and presented with lower-risk features in the clinical and laboratory findings (S1 Table). Heart failure in Killip classes 2 and 3 was less frequent in the low GRS group than in the high GRS group. The prescription of clopidogrel was notable in both low and high GRS groups. Potent P2Y inhibitors, including prasugrel and ticagrelor, were more common in the low-GRS group than in the high-GRS group. In both GRS groups, the order of predominance for IRA was LAD (left anterior descending), RCA (right coronary artery), LCX (left circumflex), and LM (left main Complex lesions (ACC/AHA type B2/C) were observed in more than 50% of cases in both groups, and significantly more prevalent in the high GRS group compared to the low GRS group. Multi-vessel disease was less prevalent in the low GRS group, and left ventricular ejection fraction (LVEF) was higher in the same group. New-onset heart failure (HF) occurred less frequently in the low GRS group. Revascularization of the infarction-related artery (IRA) occurred less frequently in the low GRS group than in the high GRS group (S1 Table).

**Table 1:** Baseline characteristics between VEIS and DIS in low (≤140) and high (>140) GRS.

Variables	Low GRS (n = 2,917)			High GRS (n = 1,816)			*P*
	VEIS (n = 1,247)	DIS (n = 1,670)	*P*	VEIS (n = 854)	DIS (n = 962)	*P*	
**Female, n (%)**	242 (19.4)	345 (20.7)	0.431	330 (38.6)	410 (42.6)	0.094	< 0.001
**Age (years)**	58.7 ± 10.6	59.6 ± 10.8	0.020	73.3 ± 8.6	73.8 ± 8.4	0.204	< 0.001
**GRACE risk score**	110.4 ± 19.9	109.9 ± 19.6	0.450	171.6 ± 28.3	169.4 ± 26.7	0.094	< 0.001
**Median PCI time (IQR) (hours)**	3.9 (2.3–6.4)	23.6 (18.0–41.8)	< 0.001	3.6 (2.1–5.7)	23.6 (18.1–51.6)	< 0.001	< 0.001
**SBP (mmHg)**	141.8 ± 25.3	143.3 ± 25.2	0.100	125.7 ± 22.1	129.5 ± 23.8	< 0.001	< 0.001
**Hypertension**	571 (45.8)	810 (48.5)	0.157	561 (65.7)	620 (64.4)	0.614	< 0.001
**Diabetes**	313 (25.1)	436 (26.1)	0.566	323 (37.8)	382 (39.6)	0.470	< 0.001
**Dyslipidemia**	145 (11.6)	228 (13.6)	0.118	64 (7.5)	103 (10.7)	0.022	< 0.001
**Smoking status**			0.069			0.125	< 0.001
**Never smoker**	394 (32.0)	569 (35.0)		445 (53.3)	540 (57.8)		
**Former smoker**	257 (20.9)	361 (22.2)		191 (22.9)	203 (21.7)		
**Current smoker**	580 (47.1)	695 (42.8)		199 (23.8)	191 (20.4)		
**Previous MI**	77 (6.2)	118 (7.1)	0.380	92 (10.8)	98 (10.2)	0.741	< 0.001
**Previous CVA**	61 (4.9)	85 (5.1)	0.859	90 (10.6)	107 (11.2)	0.720	< 0.001
**Killip, n (%)**			0.021			0.603	< 0.001
**1**	1181 (94.7)	1616 (96.8)		524 (62.0)	596 (61.4)		
**2**	57 (4.6)	47 (2.8)		160 (17.0)	164 (18.7)		
**3**	9 (0.7)	7 (0.4)		170 (21.0)	202 (19.9)		
**Serum Cr (mg/L)**	0.9 ± 0.9	1.0 ± 1.0	0.199	1.4 ± 1.6	1.4 ± 1.5	0.826	< 0.001
**eGFR (mL/min/1.73 m** ^**2**^ **)**	95.4 ± 36.1	92.5 ± 31.7	0.025	69.7 ± 42.6	66.7 ± 34.2	0.101	< 0.001
**Serum Hb (g/dL)**	14.4 ± 1.7	14.2 ± 1.8	0.001	12.5 ± 2.1	12.3 ± 2.2	0.023	< 0.001
**CK-MB (mg/dL)**	69.2 ± 92.3	45.8 ± 93.7	< 0.001	78.0 ± 104.4	44.5 ± 69.0	< 0.001	0.106
**Troponin-I (ng/mL)**	26.2 ± 50.1	15.1 ± 35.5	< 0.001	36.2 ± 72.3	16.6 ± 33.1	< 0.001	< 0.001
**TC (mg/dL)**	185.2 ± 45.1	181.3 ± 44.5	0.021	167.1 ± 46.4	167.4 ± 45.1	0.880	< 0.001
**LDL-C (mg/L)**	118.1 ± 38.8	114.8 ± 37.7	0.031	102.6 ± 39.8	103.1 ± 39.2	0.820	< 0.001
**HDL-C (mg/L)**	42.4 ± 11.2	43.1 ± 11.2	0.095	41.9 ± 12.7	42.7 ± 12.5	0.177	0.208
**NTproBNP (pg/mL)**	787.1 ± 1833.9	932.2 ± 3068.0	0.212	5762.0 ± 10902.0	5704.2 ± 8666.5	0.919	< 0.001
**HbA1c (%)**	6.6 ± 1.7	6.5 ± 1.4	0.244	6.5 ± 1.4	6.6 ± 1.3	0.374	0.864
**Discharge medication, n (%)**							
**Aspirin, n (%)**	1244 (99.8)	1669 (99.9)	0.424	852 (99.8)	962 (100.0)	0.428	1.000
**Clopidogrel, n (%)**	927 (74.4)	1326 (79.4)	0.001	700 (82.0)	863 (89.7)	< 0.001	< 0.001
**Prasugrel, n (%)**	230 (18.4)	177 (10.6)	< 0.001	70 (8.2)	51 (5.3)	0.018	< 0.001
**Ticagrelor, n (%)**	319 (35.1)	389 (31.9)	0.130	201 (33.8)	154 (22.9)	< 0.001	0.002
**CCBs, n (%)**	78 (6.2)	180 (10.8)	< 0.001	55 (6.4)	91 (9.5)	0.023	0.364
**BBs, n (%)**	1098 (88.1)	1446 (86.6)	0.265	674 (78.9)	786 (81.7)	0.152	< 0.001
**RAASis, n (%)**	1089 (87.3)	1403 (84.0)	0.014	671 (78.6)	741 (77.0)	0.463	< 0.001
**Statins, n (%)**	1209 (97.0)	1601 (95.9)	0.149	766 (89.7)	871 (90.5)	0.600	< 0.001
**OMT, n (%)**	962 (77.2)	1213 (72.6)	0.006	557 (65.4)	616 (64.0)	0.584	< 0.001
**Infarct-related artery**			0.628			0.104	< 0.001
**Left main, n (%)**	23 (1.8)	39 (2.3)		36 (4.2)	42 (4.4)		
**LAD, n (%)**	544 (43.6)	696 (41.7)		333 (39.0)	415 (43.1)		
**LCx, n (%)**	337 (27.0)	460 (27.5)		224 (26.2)	207 (21.5)		
**RCA, n (%)**	343 (27.5)	475 (28.4)		261 (30.6)	298 (31.0)		
**ACC/AHA type B2/C lesions**	1061 (85.1)	1356 (81.2)	0.007	737 (86.3)	815 (84.7)	0.375	0.007
**Extent of CAD**			0.063			0.002	< 0.001
**SVD, n (%)**	672 (53.8)	841 (50.3)		344 (40.3)	318 (33.0)		
**MVD, n (%)**	576 (46.2)	831 (49.7)		510 (59.7)	644 (66.9)		
**RVSC status**			0.121			0.013	< 0.001
**IRA only, n (%)**	282 (22.6)	419 (25.2)		288 (33.8)	380 (39.6)		
**Complete, n (%)**	965 (77.4)	1246 (74.8)		563 (66.2)	580 (60.4)		
**PCI treatment**			0.394				0.707
**Stent**	1156 (92.7)	1527 (91.4)		72 (8.4)	78 (8.1)		
**Balloon only**	89 (7.1)	138 (8.3)		1 (0.1)	5 (0.5)		
**Others**	2 (0.2)	5 (0.3)		781 (91.5)	879 (91.4)		
**Type of stent**			0.240			0.877	< 0.001
**BMS**	28 (2.4)	26 (1.7)		43 (5.5)	51 (5.8)		
**DES**	1128 (97.6)	1501 (98.3)		738 (94.5)	828 (94.2)		
**ST change, n (%)**	669 (53.6)	799 (47.8)	0.002	677 (79.3)	718 (74.6)	0.022	< 0.001
**LVEF (%)**	56.6 ± 8.8	56.6 ± 8.8	0.861	50.1 ± 11.7	49.8 ± 12.6	0.633	< 0.001
**CS, n (%)**	13 (1.0)	19 (1.1)	0.950	85 (10.0)	69 (7.2)	0.041	< 0.001
**New HF, n (%)**	11 (0.9)	8 (0.5)	0.268	63 (7.4)	78 (8.1)	0.622	< 0.001

The data were summarized as means ± SD or numbers with percentages. Statistical analyses included unpaired t-tests for continuous variables and chi-square or Fisher’s exact tests for categorical variables. GRACE, Global Registry of Acute Coronary Events; SBP, systolic blood pressure; MI, myocardial infarction; CVA, cerebrovascular accident; Cr, creatinine; Hb, hemoglobin; CK-MB, creatine kinase myocardial band; TC, total cholesterol; LDL-C, low-density lipoprotein-cholesterol; HDL-C, high-density lipoprotein-cholesterol; IQR, interquartile range; NTproBNP, N-terminal pro-brain natriuretic peptide; CCBs, calcium channel blockers; BBs, beta-blockers; RAASis, renin-angiotensin-aldosterone system inhibitors; OMT, optimal medical therapy; LAD, left anterior descending; LCx, left circumflex; RCA, right coronary artery; ACC/AHA, American College of Cardiology/ American Heart Association; CAD, coronary artery disease; SVD, single vessel disease; MVD, multi-vessel disease; RVSC, revascularization; IRA, infarction related artery; ECMO, extracorporeal membrane oxygenation; IABP, intra-aortic balloon pump: PCI, percutaneous coronary intervention; BMS, bare metal stent; DES, drug eluting stent; LVEF, left ventricular ejection fraction; CS, cardiogenic shock; HF, heart failure.

#### 3.1.1 The low GRS group

Within the low GRS group, VEIS demonstrated lower age, systolic blood pressure (SBP), serum creatinine levels, and a reduced number of CAD vessels compared to DIS, while exhibiting higher levels of creatine kinase myocardial band (CK-MB), troponin I (Tn I), total cholesterol (TC), and LDL cholesterol (LDL-C). Heart failure by Killip class did not differ between VEIS and DIS. RAAS inhibitors (RAASi) were more frequently prescribed in the VEIS group, whereas prescriptions of beta blockers (BB) and statins remained similar. Optimal medical therapy (OMT) was more commonly prescribed to the VEIS group than to the DIS. The prevalence of multi-vessel disease (MVD) did not differ between the VEIS and DIS groups. IRA-only revascularization rates were similar between the VEIS and DIS groups.

#### 3.1.2 The high GRS group

In the high GRS group, VEIS demonstrated lower SBP and MVD than DIS. However, VEIS revealed elevated levels of cardiac enzymes including creatine kinase myocardial band (CK-MB) and troponin I (TnI). HF of Killip classes 2 and 3 occurred more frequently in the high GRS group than in the low GRS group, with no significant difference between VEIS and DIS within the high GRS group. Cardiogenic shock was more frequently observed on VEIS in the high GRS group. Similar to the low GRS group, prasugrel and ticagrelor were the predominant P2Y12 inhibitors that were prescribed more frequently in the VEIS group. The distribution of RAAS inhibitors (RAASi), beta-blockers (BB), and statins was comparable in the high GRS group. The prevalence of MVD was notably higher in the high GRS group and significantly lower in the VEIS group than that in the DIS group. Revascularization of the IRA only was more common in the high GRS group, while this approach was less common in VEIS group to DIS (34% vs. 40%).

### 3.2 Clinical outcomes of very early and delayed invasive strategy

#### 3.2.1 In hospital outcomes

**Fig 2:**
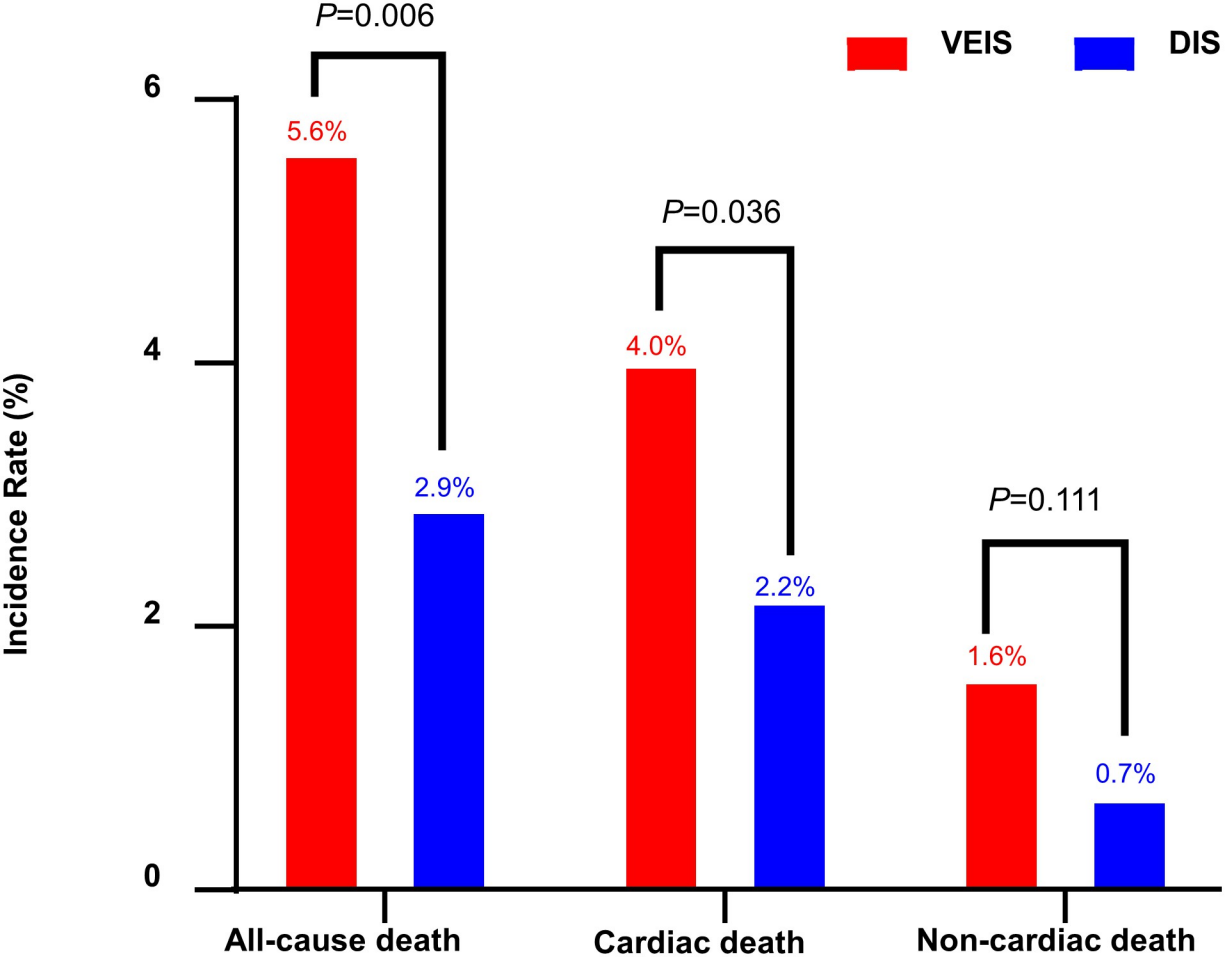
In-Hospital mortality in the high GRS group (>140).

In the low GRS group, no in-hospital mortality was reported for ACD, CD, or NCD. However, in the high GRS group, VEIS had worse clinical outcomes, with a higher incidence of in-hospital ACD (5.6% vs. 2.9%, P = 0.006) and in-hospital CD (4.0% vs. 2.2%, P = 0.036) compared to DIS (Fig 2, Table 2).

**Table 2:** In-hospital mortality between VEIS and DIS in low GRS (≤140) and high GRS (>140).

Death rate	Low GRS			High GRS		
	VEIS (n = 1,247)	DIS (n = 1,670)	*P*	VEIS (n = 854)	DIS (n = 962)	*P*
**In-hospital ACD, n (%)**	0 (0.0)	0 (0.0)	**>0.999**	48(5.6)	28 (2.9)	0.006
**In-hospital CD, n (%)**	0 (0.0)	0 (0.0)	**>0.999**	34 (4.0)	21 (2.2)	0.036
**In-hospital NCD, n (%)**	0 (0.0)	0 (0.0)	**>0.999**	14 (1.6)	7 (0.7)	0.111

Values represent n (incidence rate, %). ACD, all-cause death; CD, cardiac death; NCD, non-cardiac death; VEIS, very early invasive strategy; DIS, delayed invasive strategy; GRS, GRACE risk score

#### 3.2.2 Outcomes in 12 months

**Fig 3:**
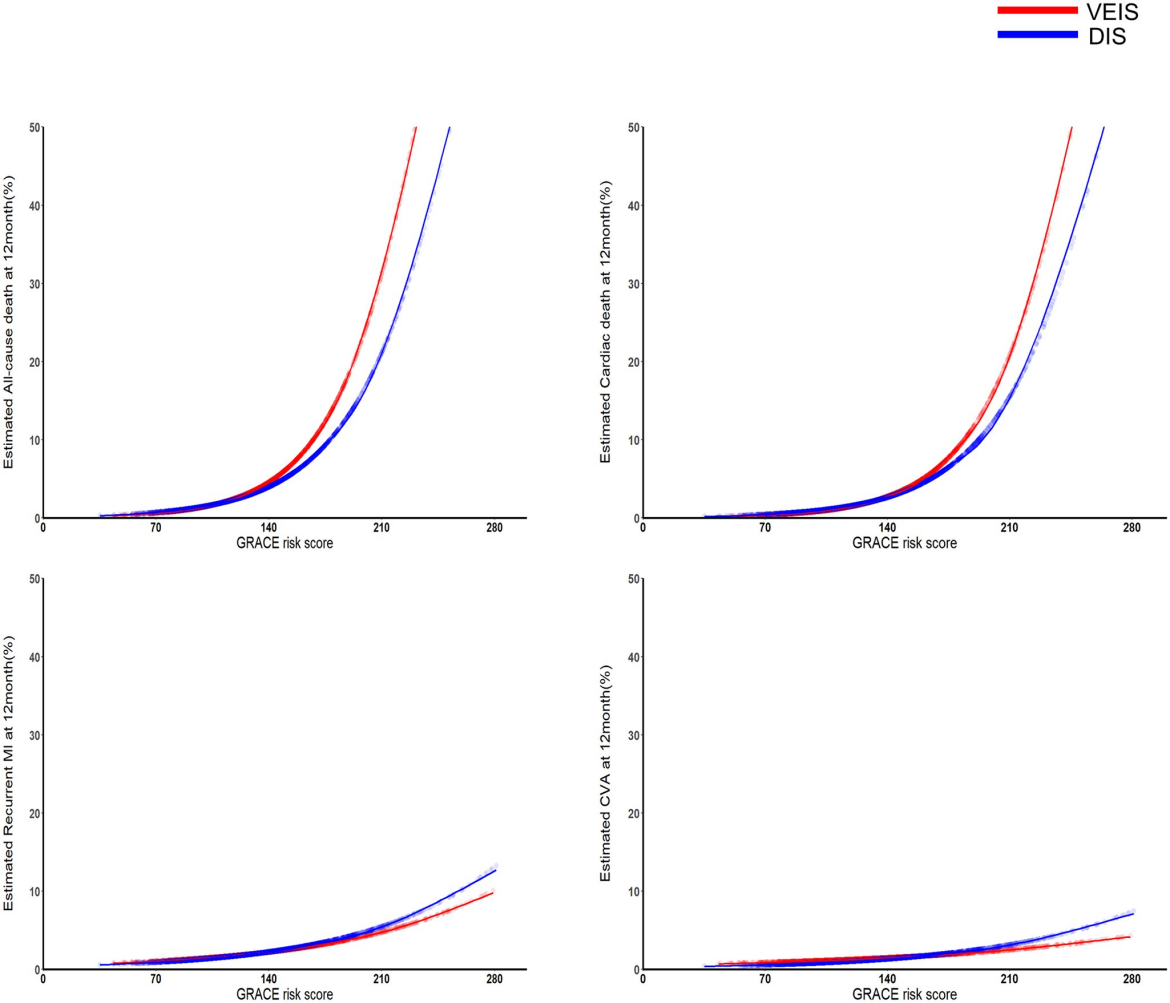
Continuous association between GRS and risk of clinical outcomes. Continuous association between GRS and risk of clinical outcomes is demonstrated according to GRS. The estimated clinical event rate was calculated from Cox proportional hazard regression model.

The association between continuous GRS and the risk of clinical events is shown in Fig 3. and S3 Table. Increasing the GRS was significantly associated with adverse clinical outcomes, including ACD (hazard ratio [HR]: 1.25 per 10 GRS increase; 95% CI: 1.20–1.29; P <0.001), CD (HR: 1.27 per 10 GRS increase; 95% CI: 1.00–1.04; P <0.001), and recurrent MI (HR: 1.09 per 10 GRS increase; 95% CI: 1.02–1.16; P = 0.014). However, regarding CVA at 12 months, increasing GRS did not elevate the risk (HR: 1.02 per 10 GRS increase; 95% CI: 0.93–1.12; P = 0.710) (S4 Table). The trend of increased estimated risk of ACD with an increase in GRS was similar for both VEIS and DIS (P for interaction = 0.056).

In the low GRS group, ACD (HR, 1.08; 0.62–1.87; P = 0.793), CD (HR, 0.95; 0.47–1.95; P = 0.895), MI (HR, 1.15; 0.65–2.04; P = 0.631), and CVA (HR, 1.15; 0.56–2.37; P = 0.694) were not different between the EIS and DIS over 12 months of follow-up (Table 3). After multivariable-adjusted analysis, the primary outcome (ACD: adjusted HR [aHR], 1.14;0.65–1.98; P = 0.648) and secondary clinical outcomes (CD: aHR 1.00; 0.49–2.04; P = 0.997; MI: aHR 1.20; 0.68–2.14; P = 0.533and CVA: 1.22; 0.59–2.52; P = 0.583) were not significantly different between the VEIS and DIS (Table 3). These results were also confirmed after 1:1 propensity score matching (PSM) analysis, indicating that the primary and secondary clinical outcomes did not exhibit significant differences between the VEIS and DIS groups (Table 3). However, in the high GRS group, ACD was significantly more frequent in VEIS (HR, 1.39; 1.08–1.78; p = 0.010). After multivariable adjustment also revealed an unfavorable outcome (aHR 1.46; 1.13–1.89; P = 0.003). The adverse impact of VEIS on ACD was confirmed even after the 1:1 PSM analysis, showing that VEIS led to worse outcomes for ACD at 12 months (HR 1.34; 1.01–1.78; P = 0.042). Regarding CD, VEIS showed a tendency towards worse outcomes relative to DIS, although this distinction did not attain statistical significance in the multivariable adjusted analysis (aHR 1.34; 0.98–1.83; P = 0.066). Notably, there were no noteworthy distinctions between VEIS and DIS in terms of recurrent MI (aHR 1.10; 0.66–1.82; P = 0.718) and CVA (aHR 0.88; 0.44–1.76; P = 0.708) after multivariable adjustment analysis (Table 3). S1 Fig shows a subgroup analysis of patients with ACD at 12 months. The results of the subgroup analysis, using a Cox logistic regression model, revealed that in all subgroups, there was a non-significant interaction regarding PCI timing, indicating an insignificant impact of subgroups on ACD rates in this study. Landmark analysis showed that incidence of ACD was greater within the first 30 days after the VEIS in the high GRS group (aHR 1.85; 1.20–2.85; P = 0.005) (S2 Fig).

**Table 3:** Clinical outcomes between VEIS and DIS at 12th month in low GRS (≤140) and high GRS (>140).

**Low risk group (GRS ≤ 140)**								
**Outcomes**	**VEIS** **(n = 1,247)**	**DIS** **(n = 1,670)**	**Unadjusted**		**Adjusted** ^a^		**Propensity score-adjusted** ^a^	
			**HR (95%CI)**	***p***	**HR (95%CI)**	***p***	**HR (95%CI)**	***p***
**All-cause death**	23 (2.1)	28 (1.8)	1.08 (0.62–1.87)	0.795	1.14 (0.65–1.98)	0.648	1.46 (0.74–2.90)	0.273
**Cardiac death**	13 (1.2)	18 (1.1)	0.95 (0.47–1.94)	0.894	1.00 (0.49–2.04)	0.997	1.29 (0.54–3.09)	0.569
**Recurrent MI**	22 (2.1)	25 (1.7)	1.15 (0.65–2.04)	0.632	1.20 (0.68–2.14)	0.533	1.20 (0.62–2.33)	0.587
**CVA**	15 (1.2)	16 (1.0)	1.15 (0.56–2.36)	0.695	1.22 (0.59–2.52)	0.583	1.02 (0.45–2.21)	0.969
**High risk group (GRS>140)**								
**Outcomes**	**VEIS** **(n = 854)**	**DIS** **(n = 962)**	**Unadjusted**		**Adjusted** ^a^		**Propensity score-adjusted** ^a^	
			**HR (95%CI)**	***p***	**HR (95%CI)**	***p***	**HR (95%CI)**	***p***
**All-cause death**	137 (16.9)	112 (12.8)	1.39 (1.08–1.78)	0.010	1.46 (1.13–1.89)	0.003	1.34 (1.01–1.78)	0.042
**Cardiac death**	87 (10.8)	78 (8.9)	1.26 (0.93–1.71)	0.134	1.34 (0.98–1.83)	0.066	1.46 (0.84–1.70)	0.328
**Recurrent MI**	29 (4.0)	33 (3.9)	1.00 (0.60–1.64)	0.986	1.10 (0.66–1.82)	0.718	1.28 (0.73–2.24)	0.386
**CVA**	16 (2.1)	20 (2.4)	0.88 (0.45–1.74)	0.723	0.88 (0.44–1.76)	0.708	0.87 (0.41–1.86)	0.722

Values represent events (Kaplan-Meier estimate, %).

^a^ Adjusted for age, sex, dyslipidemia, diabetes, extent of CAD, serum hemoglobin, optimal medical therapy, history of MI, history of CVA, and revascularization status.

CVA, cerebrovascular accident; MI, myocardial infarction; PSM, propensity score matching; Hb, hemoglobin; OMT, optimal medical treatment; CAD, coronary artery disease.

**Fig 4:**
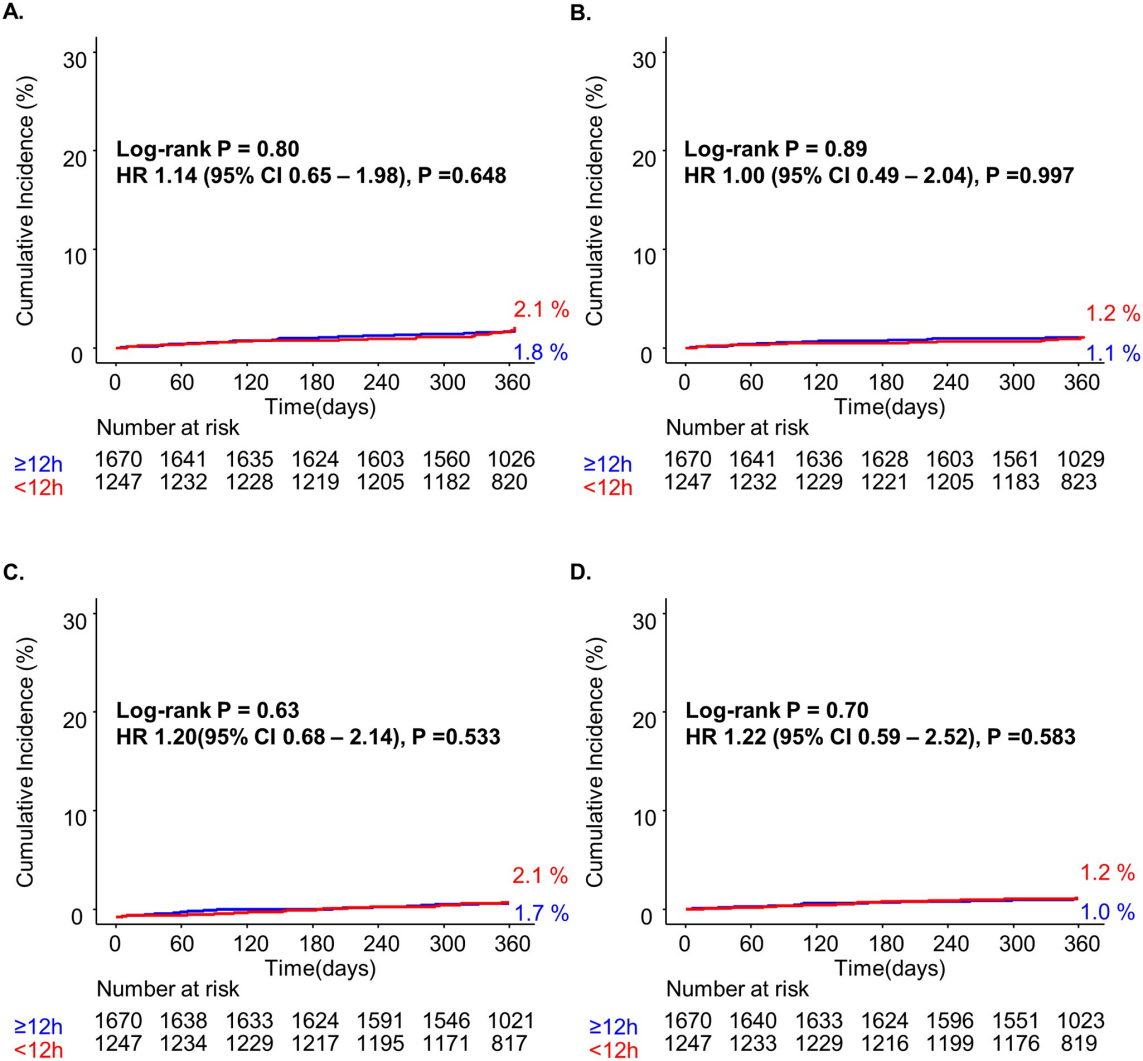
1-year clinical outcomes in low GRS (≤140) according to PCI time strategy. Kaplan-Meier analysis of all-cause death (ACD) (A), cardiac death (CD) (B), recurrent myocardial infarction (MI) (C), and cerebrovascular accident (CVA) (D) during the 12-month follow-up period in the high-risk group (GRS >140).

**Fig 5:**
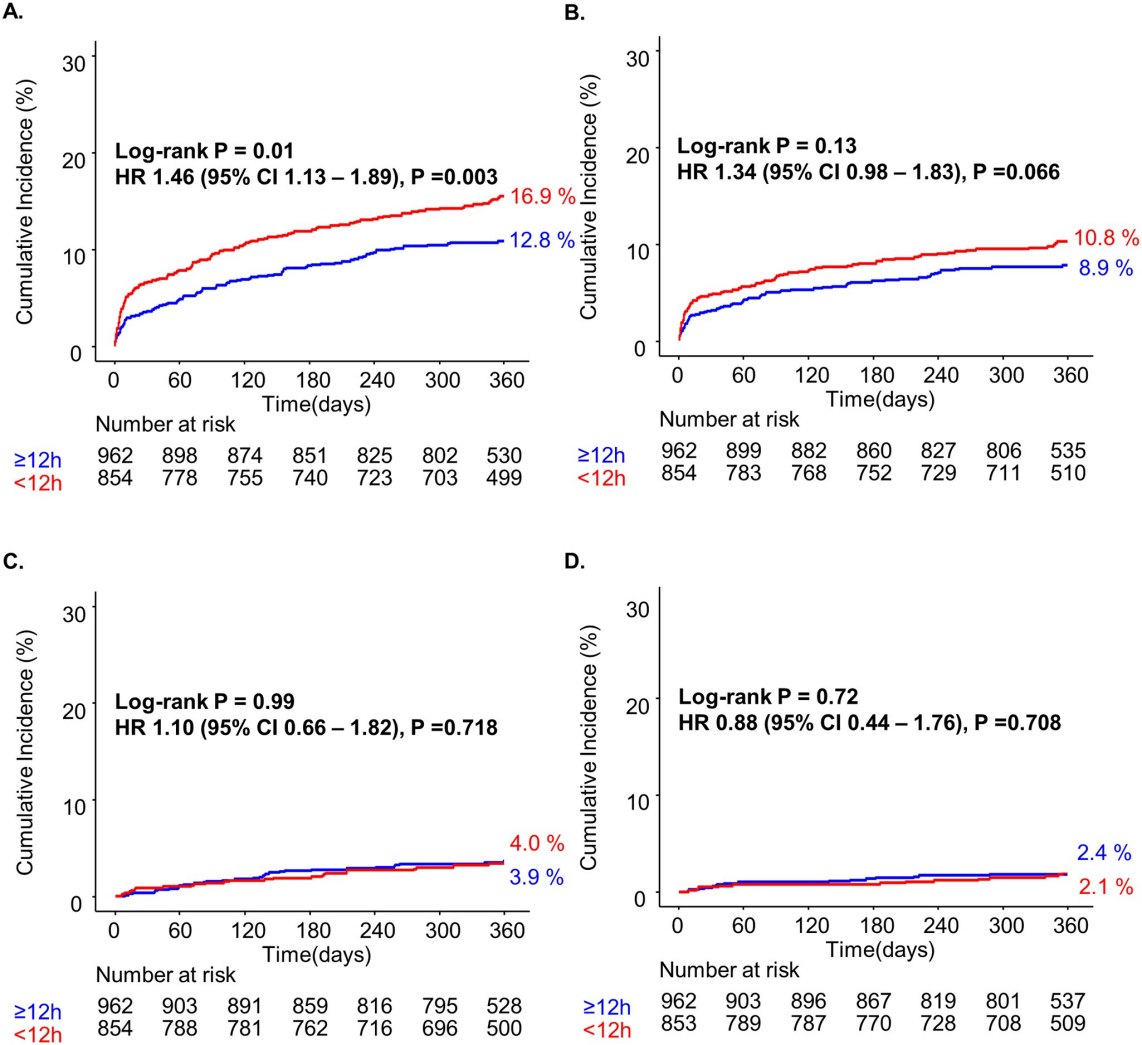
1-year clinical outcomes in high GRS (>140) according to PCI time strategy. Kaplan-Meier analysis for ACD (A), CD (B), recurrent MI (C), and CVA (D) during 12-month follow-up period in low-risk group (GRS ≤140).

Among patients in the low GRS group, no significant differences were confirmed by Kaplan–Meier (KM) analyses for all clinical endpoints (Fig 4). However, in the KM analysis of the high-GRS group, VEIS was associated with a higher probability of ACD than was DIS (P = 0.01). No significant distinction was observed between VEIS and DIS in terms of CD at 12 months (P = 0.134), although there was an inclination towards unfavorable outcomes in the VEIS group (Fig 5). The KM analysis when stratified into three groups according to 12-hour intervals showed no significant difference (S3 Fig).

## 4. Discussion

In this prospective, observational study, we retrospectively investigated the clinical outcomes of VEIS and DIS in patients with NSTEMI without hemodynamic instability at the time of admission, stratified by high or low GRS, using KAMIR. The main findings of this study are as follows:1) VEIS and DIS showed comparable outcomes in patients with NSTEMI and a low GRS. 2) Among NSTEMI patients with a high GRS, VEIS resulted in worse outcomes than DIS in terms of ACD and CD. 3) Independent predictors of ACD at 12 months included VEIS, new-onset HF, revascularization status, serum hemoglobin level, optimal medical therapy, dyslipidemia, and extent of coronary artery disease. To the best of our knowledge, this study is the first to demonstrate unfavorable outcomes associated with VEIS compared to DIS in a high-risk NSTEMI patient group, while excluding situations requiring urgent PCI in real clinical practice.

Early revascularization offers the benefit of promptly identifying obstructive coronary lesions and effectively alleviating the myocardial ischemic burden in patients with NSTEMI [11, 13]. Specifically, early revascularization may be more effective in NSTEMI patients with an unstable hemodynamic status associated with cardiac collapse. However, this strategy has inherent limitations. This may restrict the time available for medical interventions aimed at dissolving thrombi, which could result in myocardial embolic damage during PCI [11]. Furthermore, it may reduce the opportunity for implementing effective pretreatment to prevent acute kidney injury and HF conditions that can be exacerbated by unprotected PCI in patients with NSTEMI [14, 15]. Therefore, determining the optimal timing for NSTEMI requires meticulous assessment of each patient’s risk profile.

We found that VEIS yielded outcomes comparable to those of DIS in patients with NSTEMI and a low-risk profile (GRS < 140). However, in patients with NSTEMI with a high-risk profile and no hemodynamic instability, VEIS demonstrated inferior results compared to DIS. In patients with high GRS, the clinical events associated with VEIS were notable, particularly evident in in-hospital outcomes, as indicated by higher rates of ACD (5.6% vs. 2.9%, P = 0.006) and CD (4.0% vs. 2.2%, P = 0.036). The discrepant results observed between our study and earlier trials focusing on high-risk NSTEMI cases may be attributed to variations in the extent of specific GRS factors in the absence of significant hemodynamic conditions. The GRS encompasses eight independent clinical variables: age, heart rate, systolic blood pressure, serum creatinine level, Killip class, cardiac arrest upon admission, elevated cardiac markers, and ST-segment deviation upon arrival. We excluded very high-risk cases with an initial SBP of < 90 mmHg, ventricular arrhythmia, and Killip IV heart failure from the GRS calculation, leading to a system in which variables such as age and serum creatinine level carry a relatively higher weight in the GRS. Patients with a high GRS within this system may have an elevated relative risk in the context of unprotected PCI without adequate pretreatment. Recent reports have indicated that EIS within 24hours was not associated with clinical advantages in older age [13]. Another report demonstrated that EIS and DIS yielded similar clinical outcomes in NSTEMI patients with CKD [16]. These reports support the idea that elderly patients or patients with renal dysfunction might benefit from delayed PCI with appropriate pretreatment. Moreover, the impact of GRS on NSTEMI clinical outcomes may vary depending on ethnic diversity. The GRS is a validated risk assessment tool based on clinical variables upon arrival, and was developed using data from the GRACE registry, which included 20,000 patients with acute coronary syndrome (ACS) from 94 hospitals in 14 countries across Europe, North and South America, Australia, and New Zealand between 1999 and 2003 [17]. A recent study revealed variations in the predictive accuracy of the GRS for in-hospital mortality in patients with NSTEMI between Caucasians and ethnic minorities including Asians [19]. This disparity could contribute to the divergent outcomes observed in our study compared to previous trials. Another significant finding of our study was the similarity in the distribution of VEIS and DIS proportions within both the low (43% vs. 57%) and high (47% vs. 53%) GRS groups. Furthermore, when analyzed based on a 24-hour PCI time criterion, both the low and high GRS groups exhibited an identical ratio of EIS (<24h) to DIS (≥24h) at 72% to 28%. This suggests that despite guideline recommendations, GRS might not have been the foremost factor guiding clinicians’ decisions regarding the timing of NSTEMI treatment in real-world practice. Hansen et al. noted that in contemporary real-world practice, the EIS pattern tends to be delayed for NSTEMI patients with risk factors such as advanced age, CKD, and HF on admission, components of high-risk features that are typically indicated for early PCI [18].

Since its introduction, advancements have been made in the treatment ACS over the intervening period. Our data were collected between 2011 and 2015, a time of transition to DAPT involving more potent P2Y12 inhibitors such as ticagrelor and prasugrel in the treatment of ACS as well as the predominant utilization of second-generation drug-eluting stents in PCI. In our study, aspirin combined with clopidogrel was preferred for DAPT over other potent P2Y12 inhibitors for DAPT. A recent study examining the influence of GRS on the treatment and outcomes of patients admitted for NSTEMI revealed that although the incorporation of routine GRS led to a higher utilization of EIS, it did not significantly reduce patient mortality compared to the control group [19]. Given that contemporary treatment has advanced, these findings underscore the importance of adopting a personalized approach, distinct from the GRS, to identify patients with NSTEMI who could potentially benefit from an optimal invasive time strategy. In addition, the impact of the GRS on NSTEMI clinical outcomes may vary depending on ethnic diversity. A recent study revealed variations in the predictive accuracy of the GRS for in-hospital mortality in patients with NSTEMI between Caucasians and ethnic minorities including Asians [20]. This disparity may have contributed to the different results observed in our study compared with those of previous trials.

### Limitation

Our study has several limitations that warrant consideration. First, we excluded patients at very high risk due to hemodynamic instability resulting from conditions such as shock, arrhythmia, or Killip class 4 heart failure. Second, our analysis focused specifically on NSTEMI patients who underwent PCI following CAG, thereby excluding those who underwent surgical revascularization or received only medical treatment—groups that might be at higher risk. This exclusion could introduce selection bias. Third, our analysis was confined to East Asian populations. As a result, caution should be exercised when extrapolating these findings to other ethnic or geographical groups, as they may not be universally applicable. Fourth, the registry lacked data on periprocedural complications, including periprocedural infarction, no-reflow phenomenon, contrast-induced nephropathy, and bleeding. The absence of such specific details is a limitation, given that these complications could significantly impact adverse clinical outcomes. Consequently, future studies should incorporate comprehensive data on periprocedural complications to provide a more nuanced analysis of the clinical implications.

## Conclusion

In this analysis of a real-world cohort of patients with NSTEMI without hemodynamic instability, VEIS demonstrated comparable outcomes to DIS for patients with a low GRS at the 12-month follow-up. However, among patients with a high GRS, VEIS resulted in worse outcomes over the 12-month period than DIS. Additional randomized studies are required to validate these findings.

## Supporting information

S1 Fig Subgroup analysis of all-cause death at 12 month. (TIF)

S2 Fig 30-day landmark analysis in high GRS (>140) for all-cause death and cardiac death at 12 month. (TIF)

S3 Fig 1-year clinical outcomes in high GRS (>140) according to PCI time strategy. (TIF)

S1 Table Baseline characteristics between the low (≤140) and high (>140) GRS group. (DOCX)

S2 Table Baseline characteristics of the 1:1 PS-matched population in low GRS (≤140). (DOCX)

S3 Table Baseline characteristics of the 1:1 PS-matched population in high GRS (>140). (DOCX)

S4 Table The association between continuous GRACE score and the risk of clinical events at 12 month. (DOCX)

## Abbreviations

DIS: Delayed invasive strategy
GRACE: Global Registry of Acute Coronary Event
NSTEMI: Non-ST segment elevation myocardial infarction
PCI: Percutaneous coronary intervention
VEIS: Very early invasive strategy
