# Supplementary figures and images for: Very early vs delayed invasive strategy in high-risk NSTEMI patients without hemodynamic instability: Insight from the KAMIR-NIH
Source: PLoS One. 2024 Jun 6;19(6):e0304273. doi: 10.1371/journal.pone.0304273 (PMC11156373; doi:10.1371/journal.pone.0304273)

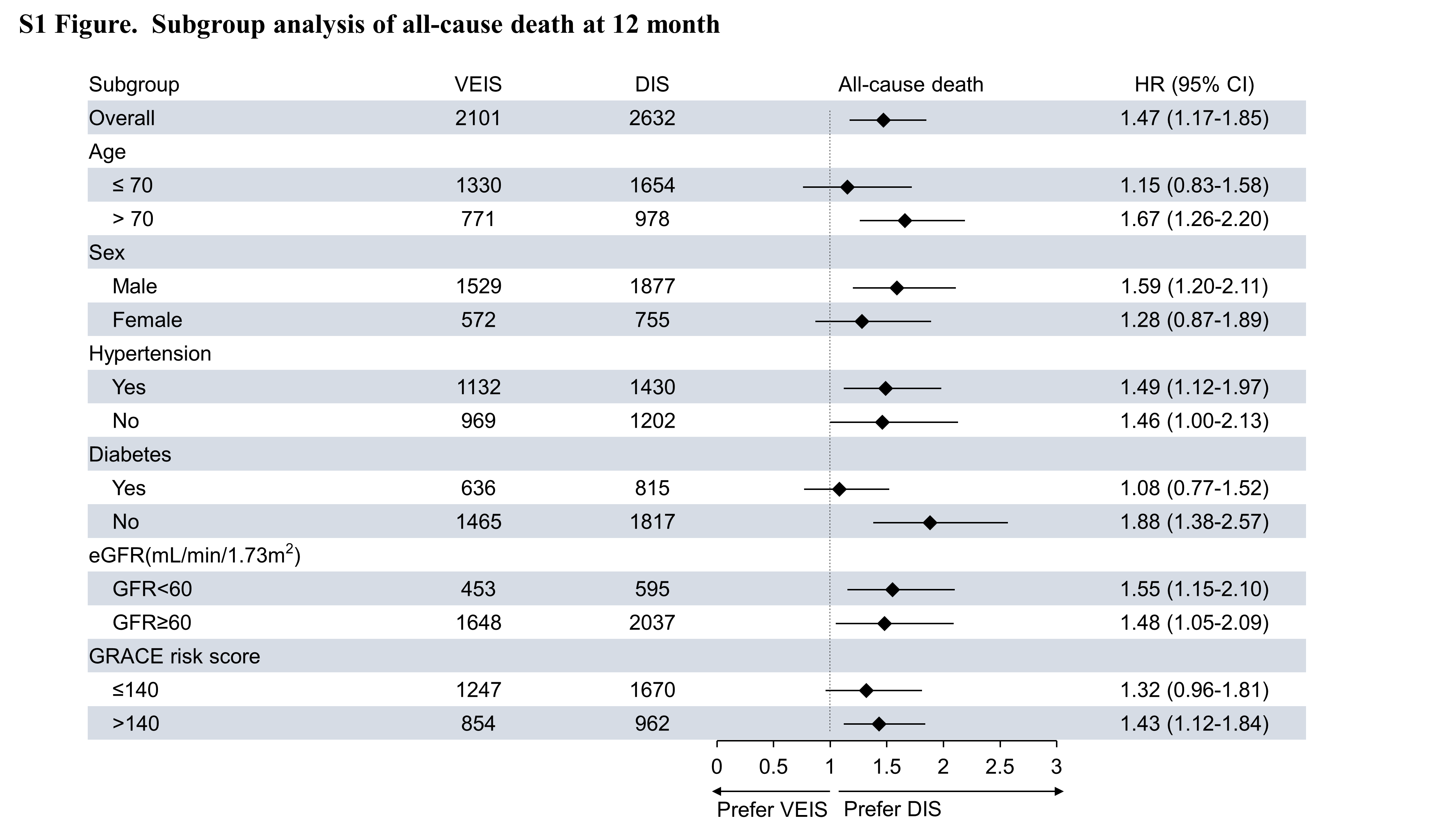

Supplement: S1 Fig — (TIF) [file pone.0304273.s001.tif]

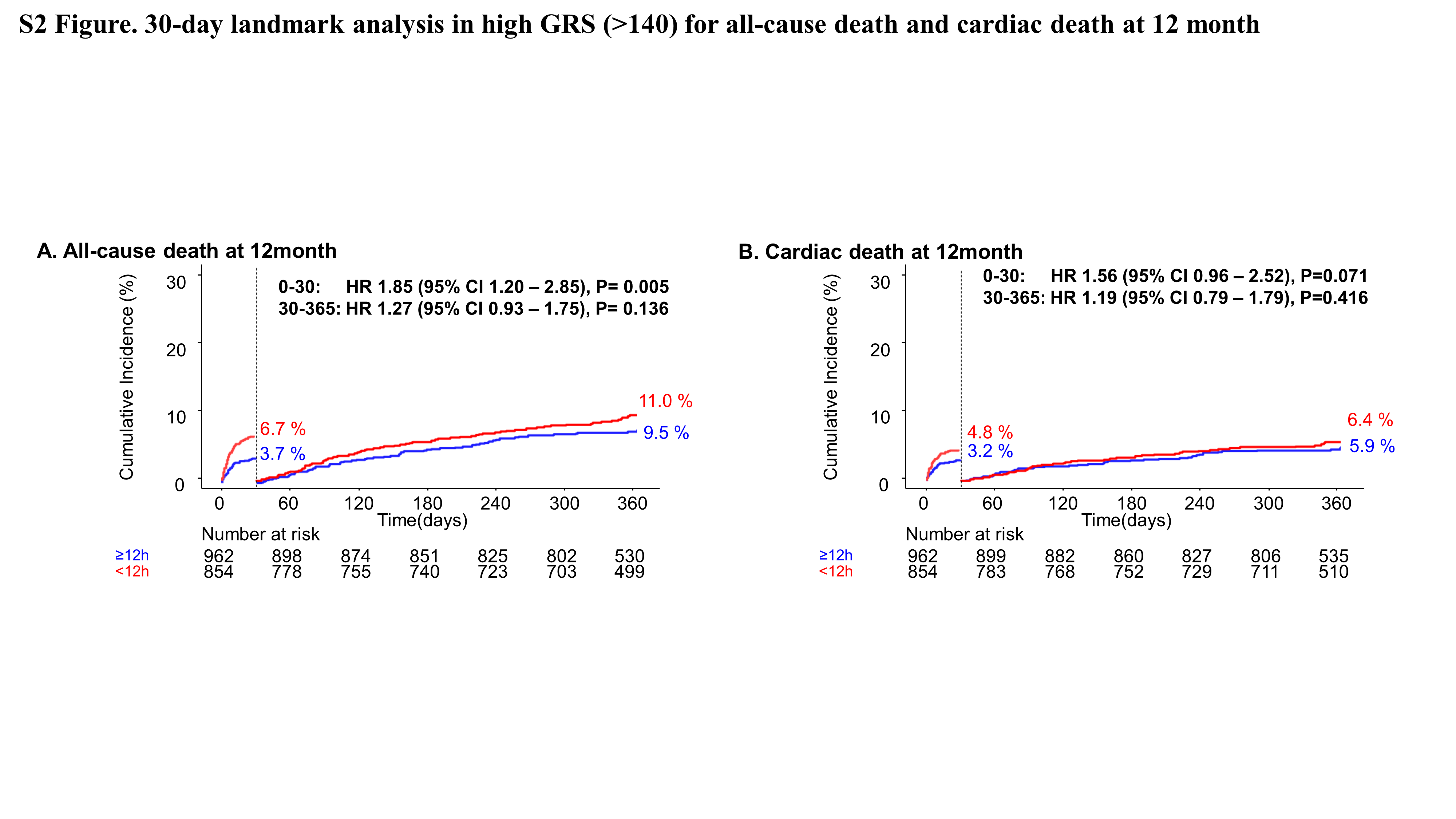

Supplement: S2 Fig — (TIF) [file pone.0304273.s002.tif]

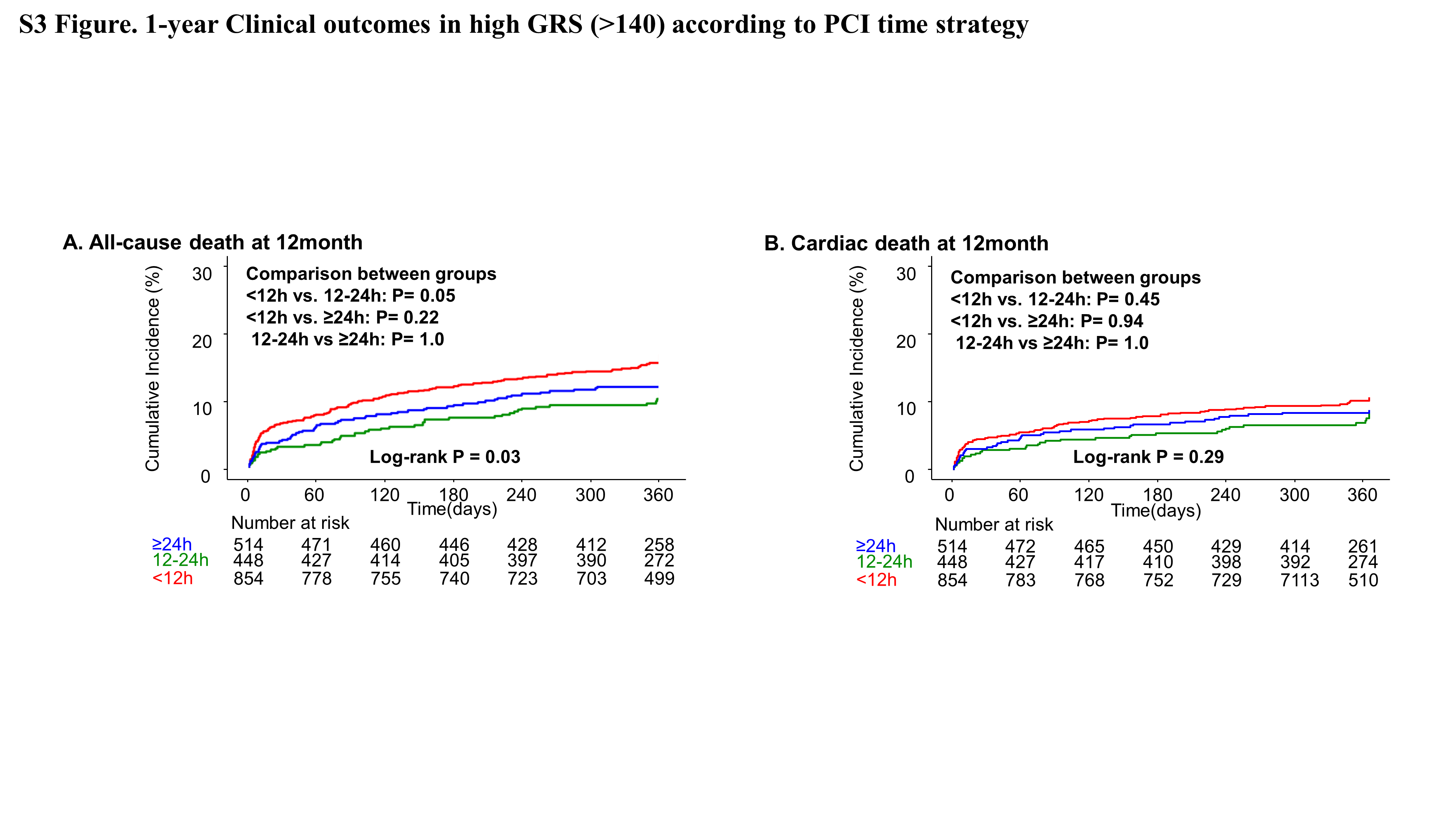

Supplement: S3 Fig — (TIF) [file pone.0304273.s003.tif]
