# Supplementary material for: Very early vs delayed invasive strategy in high-risk NSTEMI patients without hemodynamic instability: Insight from the KAMIR-NIH
Source: PLoS One. 2024 Jun 6;19(6):e0304273. doi: 10.1371/journal.pone.0304273 (PMC11156373; doi:10.1371/journal.pone.0304273)
Supplement: S1 Table — (DOCX) [file pone.0304273.s004.docx]

**S1 Table. Baseline characteristics between the low (≤140) and high (>140) GRS group**

|  | **Low GRS**  **(n=2,917)** | **High GRS**  **(n=1,816)** | ***P*** |
| --- | --- | --- | --- |
| **Female, n (%)** | 587 (20.1) | 740 (40.7) | < 0.001 |
| **Age (years)** | 59.2 ± 10.7 | 73.6 ± 8.5 | < 0.001 |
| **GRACE risk score** | 110.1 ± 19.7 | 170.4 ± 27.5 | < 0.001 |
| **Median PCI time (IQR) (hours)** | 15.4 (18.0-41.8) | 13.7 (3.8-6-27.0) | 0.123 |
| **SBP (mmHg)** | 142.7 ± 25.2 | 127.7 ± 23.1 | < 0.001 |
| **Hypertension** | 1381 (47.3) | 1181 (65.0) | < 0.001 |
| **Diabetes** | 749 (25.7) | 702 (38.7) | < 0.001 |
| **Dyslipidemia** | 373 (12.8) | 167 (9.2) | < 0.001 |
| **Smoking status** |  |  | < 0.001 |
| **Never smoker** | 963 (33.7) | 985 (55.7) |  |
| **Former smoker** | 618 (21.6) | 394 (22.3) |  |
| **Current smoker** | 1275 (44.6) | 390 (22.0) |  |
| **Previous MI** | 195 (6.7) | 190 (10.5) | < 0.001 |
| **Previous CVA** | 146 (5.0) | 197 (10.9) | < 0.001 |
| **Killip, n (%)** |  |  | < 0.001 |
| **1** | 2797 (95.9) | 1120 (61.7) |  |
| **2** | 104 (3.6) | 324 (17.8) |  |
| **3** | 16 (0.5) | 372 (20.5) |  |
| **Serum Cr (mg/L)** | 1.0 ± 0.9 | 1.4 ± 1.5 | < 0.001 |
| **eGFR (mL/min/1.73 m²)** | 93.8 ± 33.7 | 68.2 ± 38.3 | < 0.001 |
| **Serum Hb (g/dL)** | 14.3 ± 1.8 | 12.4 ± 2.2 | < 0.001 |
| **CK-MB (mg/dL)** | 55.8 ± 93.9 | 60.2 ± 89.0 | 0.107 |
| **Troponin-I (ng/mL)** | 19.9 ± 42.7 | 25.8 ± 55.9 | < 0.001 |
| **TC (mg/dL)** | 183.0 ± 44.8 | 167.3 ± 45.7 | < 0.001 |
| **LDL-C (mg/L)** | 116.2 ± 38.2 | 102.9 ± 39.5 | < 0.001 |
| **HDL-C (mg/L)** | 42.8 ± 11.2 | 42.3 ± 12.6 | 0.208 |
| **NTproBNP (pg/mL)** | 868.6 ± 2600.3 | 5731.3 ± 9771.3 | < 0.001 |
| **HbA1c (%)** | 6.5 ± 1.5 | 6.5 ± 1.4 | 0.871 |
| **Medication, n (%)** |  |  |  |
| **Aspirin, n (%)** | 2913 (99.9) | 1814 (99.9) | 1.000 |
| **Clopidogrel, n (%)** | 2253 (77.2) | 1563 (86.1) | < 0.001 |
| **Prasugrel, n (%)** | 407 (14.0) | 121 (6.7) | < 0.001 |
| **Ticagrelor, n (%)** | 708 (33.3) | 355 (28.0) | 0.002 |
| **CCBs, n (%)** | 258 (8.8) | 146 (8.0) | 0.363 |
| **BBs, n (%)** | 2544 (87.2) | 1460 (80.4) | < 0.001 |
| **RAASis, n (%)** | 2492 (85.4) | 1412 (77.8) | < 0.001 |
| **Statins, n (%)** | 2810 (96.3) | 1637 (90.1) | < 0.001 |
| **OMT, n (%)** | 2175 (74.6) | 1173 (64.7) | < 0.001 |
| **Infarct-related artery** |  |  | < 0.001 |
| **Left main, n (%)** | 62 (2.1) | 78 (4.3) |  |
| **LAD, n (%)** | 1240 (42.5) | 748 (41.2) |  |
| **LCx, n (%)** | 797 (27.3) | 431 (23.7) |  |
| **RCA, n (%)** | 818 (28.0) | 559 (30.8) |  |
| **ACC/AHA type B2/C** | 2417 (82.9) | 1552 (85.5) | 0.007 |
| **Extent of CAD** |  |  | < 0.001 |
| **SVD, n (%)** | 1513 (51.9) | 662 (36.5) |  |
| **MVD, n (%)** | 1405 (48.1) | 1154 (63.5) |  |
| **RVSC status** |  |  | < 0.001 |
| **IRA only, n (%)** | 701 (24.1) | 668 (36.9) |  |
| **Complete, n (%)** | 2211 (75.9) | 1143 (63.1) |  |
| **PCI treatment** |  |  | 0.707 |
| **Stent** | 2683 (92.0) | 1660 (91.4) |  |
| **Balloon only** | 227 (7.8) | 150 (8.3) |  |
| **Others** | 7 (0.2) | 6 (0.3) |  |
| **Type of stent** |  |  | < 0.001 |
| **BMS** | 54 (2.0) | 94 (5.7) |  |
| **1^st^ Generation DES** | 48 (1.8) | 13 (0.8) |  |
| **2^nd^generation DES** | 2581 (96.2) | 1553 (93.6) |  |
| **ST change, n (%)** | 1468 (50.3) | 1395 (76.8) | < 0.001 |
| **LVEF (%)** | 56.6 ± 8.8 | 49.9 ± 12.2 | < 0.001 |
| **CS, n (%)** | 32 (1.1%) | 154 (8.5) | < 0.001 |
| **New HF, n (%)** | 19 (0.7) | 141 (7.8) | < 0.001 |
