# Supplementary material for: Very early vs delayed invasive strategy in high-risk NSTEMI patients without hemodynamic instability: Insight from the KAMIR-NIH
Source: PLoS One. 2024 Jun 6;19(6):e0304273. doi: 10.1371/journal.pone.0304273 (PMC11156373; doi:10.1371/journal.pone.0304273)
Supplement: S2 Table — (DOCX) [file pone.0304273.s005.docx]

**S2 Table. Baseline characteristics of the 1:1 PS-matched population in low GRS (≤140)**

|  | **Before PSM** | | | **After 1:1 PSM** | | |
| --- | --- | --- | --- | --- | --- | --- |
| **Variables** | **VIES (n=1,247)** | **DIS**  **(n=1,670)** | ***P*** | **VEIS (n=1,163)** | **DIS (n=1,163)** | ***P*** |
| **Killip** |  |  | 0.021 |  |  | 0.677 |
| **1** | 1181 (94.7) | 1616 (96.8) |  | 1116 (96.0) | 1118 (96.1) |  |
| **2** | 57 (4.6) | 47 (2.8) |  | 44 (3.8) | 39 (3.4) |  |
| **3** | 9 (0.7) | 7 (0.4) |  | 3 (0.3) | 5 (0.4) |  |
| **Sex (Female)** | 242 (19.4) | 345 (20.6) | 0.436 | 224 (19.3) | 213 (18.31) | 0.596 |
| **Age (years)** | 58.7 ± 10.6 | 59.6 ± 10.8 | 0.020 | 58.7 ± 10.6 | 58.6 ± 10.6 | 0.967 |
| **Systolic blood pressure (mmHg)** | 141.8 ± 25.3 | 143.4 ± 25.1 | 0.100 | 141.8 ± 25.3 | 141.5 ± 24.1 | 0.746 |
| **Diabetes** | 313 (25.1) | 436 (26.1) | 0.566 | 289 (24.9) | 283 (24.3) | 0.810 |
| **Serum hemoglobin (g/dL)** | 14.4 ± 1.7 | 14.2 ± 1.8 | 0.001 | 14.5 ± 1.7 | 14.4 ± 1.7 | 0.731 |
| **Dyslipidemia** | 145 (11.6) | 228 (13.6) | 0.119 | 137 (11.8) | 131 (11.3) | 0.745 |
| **Serum creatinine (mg/dL)** | 0.9 ± 0.9 | 1.0 ± 1.0 | 0.199 | 0.9 ± 0.8 | 0.9 ± 0.8 | 0.880 |
| **Smoking status** |  |  | 0.066 |  |  | 0.886 |
| **Never smoker** | 394 (32.0) | 569 (35.0) |  | 373 (32.1) | 362 (31.1) |  |
| **Former smoker** | 257 (20.9) | 361 (22.2) |  | 240 (20.6) | 244 (21.0) |  |
| **Current smoker** | 580 (47.1) | 696 (42.8) |  | 550 (47.3) | 557 (47.9) |  |
| **Previous MI** | 77 (6.2) | 118 (7.1) | 0.380 | 69 (5.9) | 71 (6.1) | 0.931 |
| **Previous CVA** | 61 (4.9) | 85 (5.1%) | 0.859 | 56 (4.8) | 55 (4.7) | 1.000 |
| **Optimal medical therapy** | 962 (77.2) | 1213 (72.6) | 0.006 | 898 (77.2) | 889 (76.4) | 0.694 |
| **Revascularization status** |  |  | 0.128 |  |  | 0.727 |
| **Partial revascularization** | 282 (22.6) | 457 (27.3) |  | 262 (22.5) | 254 (21.8) |  |
| **Total revascularization** | 965 (77.4) | 1213 (72.7) |  | 901 (77.5) | 909 (78.2) |  |
| **LVEF (%)** | 56.6 ± 8.8 | 56.6 ± 8.8 | 0.861 | 56.6 ± 8.8 | 56.5 ± 8.7 | 0.664 |
| **Extent of CAD** |  |  | 0.060 |  |  | 0.901 |
| **Single-vessel disease** | 672 (53.9) | 840 (50.3) |  | 625 (53.7) | 629 (54.1) |  |
| **Multi-vessel disease** | 576 (46.1) | 830 (49.7) |  | 538 (46.3) | 534 (45.9) |  |
