# Supplementary material for: Very early vs delayed invasive strategy in high-risk NSTEMI patients without hemodynamic instability: Insight from the KAMIR-NIH
Source: PLoS One. 2024 Jun 6;19(6):e0304273. doi: 10.1371/journal.pone.0304273 (PMC11156373; doi:10.1371/journal.pone.0304273)
Supplement: S3 Table — (DOCX) [file pone.0304273.s006.docx]

**S3 Table. Baseline characteristics of the 1:1 PS-matched population in high GRS (>140)**

|  | **Before PSM** | | | **After 1:1 PSM** | | |
| --- | --- | --- | --- | --- | --- | --- |
| **Variables** | **VIES**  **(n=854)** | **DIS**  **(n=962)** | ***P*** | **VEIS**  **(n=747)** | **DIS**  **(n=747)** | ***P*** |
| **Killip** |  |  | 0.603 |  |  | 0.533 |
| **1** | 524 (61.4) | 596 (62.0) |  | 454 (60.8) | 455 (60.9) |  |
| **2** | 160 (18.7) | 164 (17.0) |  | 144 (19.3) | 130 (17.4) |  |
| **3** | 170 (19.9) | 202 (21.0) |  | 149 (19.9) | 162 (21.7) |  |
| **Sex (Female)** | 330 (38.6) | 410 (42.6) | 0.094 | 299 (40.0) | 312 (41.8) | 0.528 |
| **Age (years)** | 73.3 ± 8.6 | 73.8 ± 8.4 | 0.204 | 73.4 ± 8.4 | 73.6 ± 8.5 | 0.767 |
| **SBP (mmHg)** | 125.7 ± 22.1 | 129.5 ± 23.8 | < .001 | 125.9 ± 21.9 | 126.3 ± 21.4 | 0.730 |
| **Diabetes** | 323 (37.8) | 379 (39.4) | 0.522 | 282 (37.8) | 274 (36.7) | 0.708 |
| **Serum hemoglobin (g/dL)** | 12.5 ± 2.1 | 12.3 ± 2.2 | 0.023 | 12.5 ± 2.1 | 12.5 ± 2.2 | 0.678 |
| **Dyslipidemia** | 64 (7.5) | 103 (10.7) | 0.022 | 56 (7.5) | 52 (7.0) | 0.764 |
| **Serum creatinine (mg/dL)** | 1.4 ± 1.6 | 1.4 ± 1.5 | 0.826 | 1.4 ± 1.5 | 1.4 ± 1.5 | 0.964 |
| **Smoking status** |  |  | 0.125 |  |  | 0.851 |
| **Never smoker** | 445 (53.3) | 542 (57.8) |  | 401 (55.7) | 411 (55.0) |  |
| **Former smoker** | 191 (22.9) | 203 (21.7) |  | 171 (22.9) | 169 (22.6) |  |
| **Current smoker** | 199 (23.8) | 191 (20.4) |  | 175 (23.4) | 167 (22.45) |  |
| **Previous MI** | 92 (10.8) | 98 (10.2) | 0.741 | 80 (10.7) | 77 (10.3) | 0.866 |
| **Previous CVA** | 90 (10.6) | 107 (11.2) | 0.720 | 78 (10.4) | 76 (10.2) | 0.932 |
| **Optimal medical therapy** | 557 (65.4) | 616 (64.0) | 0.584 | 499 (66.8) | 497 (66.5) | 0.956 |
| **Revascularization status** |  |  | 0.013 |  |  | 0.744 |
| **Partial revascularization** | 288 (33.8) | 380 (39.6) |  | 256 (34.3) | 263 (35.2) |  |
| **Total revascularization** | 563 (66.2) | 580 (60.4) |  | 491 (65.7) | 484 (64.8) |  |
| **LVEF (%)** | 50.1 ± 11.7 | 49.8 ± 12.6 | 0.633 | 49.9 ± 11.8 | 49.8 ± 12.8 | 0.777 |
| **Extent of CAD** |  |  | 0.002 |  |  | 0.523 |
| **Single-vessel disease** | 344 (40.3) | 318 (33.1) |  | 291 (39.0) | 278 (37.2) |  |
| **Multi-vessel disease** | 510 (59.7) | 644 (66.9) |  | 456 (61.0) | 469 (62.8) |  |
