# Supplementary material for: Very early vs delayed invasive strategy in high-risk NSTEMI patients without hemodynamic instability: Insight from the KAMIR-NIH
Source: PLoS One. 2024 Jun 6;19(6):e0304273. doi: 10.1371/journal.pone.0304273 (PMC11156373; doi:10.1371/journal.pone.0304273)
Supplement: S4 Table — (DOCX) [file pone.0304273.s007.docx]

**S4 Table.** **The association between continuous GRACE score and the risk of clinical events at 12 month**

|  | Univariate HR^*^  (95% CI) | *P* | Multivariate^†^ HR^*^  (95% CI) | *P* |
| --- | --- | --- | --- | --- |
| All-cause death | 1.32 (1.29-1.35) | <0.001 | 1.25 (1.20-1.29) | <0.001 |
| Cardiac death | 1.33 (1.29-1.37) | <0.001 | 1.27 (1.00-1.04) | <0.001 |
| Recurrent MI | 1.13 (1.08-1.19) | <0.001 | 1.09 (1.02-1.16) | 0.014 |
| CVA | 1.11 (1.05-1.18) | <0.001 | 1.02 (0.93-1.12) | 0.710 |

* Per GRACE score decrease by 10

† Adjusted by age, sex, dyslipidemia, diabetes, extent of CAD, serum hemoglobin, optimal medical therapy, history of MI, history of CVA, and revascularization status
